# Supplementary material for: Are carbon emissions trading and green financial instruments synergistic? -Comprehensive quantitative research based on content analysis
Source: PLoS One. 2024 Mar 7;19(3):e0298601. doi: 10.1371/journal.pone.0298601 (PMC10919601; doi:10.1371/journal.pone.0298601)
Supplement: S1 Data — (ZIP) [file pone.0298601.s001.zip › Data/地级市Z_F工作报告.zip!/╣π╢1⁄2/╓╨╔╜╩╨/└┤╫╘╣1⁄2╓┌║┼í╛┬φ┐╦╩2╛▌═°í┐_2015.docx]

2014年工作回顾

　　2014年，面对复杂多变的形势，特别是经济下行的压力，市政府在省委、省政府和市委的正确领导下，在市人大和市政协的监督支持下，全面贯彻党的十八大、十八届三中、四中全会和习近平总书记系列重要讲话精神，紧紧围绕“一改两稳三确保”的工作重点，团结和依靠全市人民，积极应对严峻形势的挑战，坚持稳中求进、改革创新，以实干的精神、坚定的信心，加快产业升级与城市转型,加强生态建设与民生保障，促进依法行政与社会和谐，实现了经济社会持续稳步发展，基本完成市十四届人大四次会议确定的主要目标任务。

　　（一）经济发展稳中有进。2014年，全市实现生产总值2823亿元，比上年增长8%，增速逐季提升。人均生产总值8.9万元，增长7.1%。规模以上工业增加值1312.4亿元，增长10%。服务业增加值1192.2亿元，增长7.8%。一般公共预算收入251.6亿元，增长11.6%。固定资产投资903.7亿元，增长15%。社会消费品零售总额981.8亿元，增长10%。出口总值278.8亿美元，增长5.3%。实际利用外资6.8亿美元，增长5.3%。成立“马上办”办公室，有力地推动了项目落地。实施《珠三角规划纲要》情况良好，各项重大项目进展顺利。

　　（二）经济质量稳步提升。三次产业结构调整为2.5：55.3：42.2，轻重工业比为54.8：45.2。规模以上工业劳动生产率增长11.5%。新增规上限上企业626家，净增377家。百亿级企业增至9家，十亿级企业超100家，千亿级产业集群2个。一般贸易出口占出口比重达45.6%，创历史新高。现代服务业增加值占服务业增加值比重为58.8%。承接服务外包离岸执行金额增长179.1%，增速排全省第一。金融业增加值增长10%，境内外上市挂牌公司增至27家。建成专业镇金融超市与服务平台。“助保贷”政策撬动金融机构贷款16.6亿元。大涌专业镇产业转型升级试点成效初显。启动4个市级产业平台建设。成功打造6个现代农业示范园区。研发经费占生产总值比例为2.4%，发明专利授权量增长8.8%。工业技改投资增长66.1%。主要污染物总量减排考核获省“优秀”等次，重金属污染综合防治考核、环保责任考核均排全省第二。

　　（三）改革开放步伐加快。公布行政权责“三张清单”，取消、调整各类权限173项，赋予火炬开发区、翠亨新区、小榄镇、三乡镇更大权限。启动“三规合一”规划管理体制改革。深入推进项目投资审批体制改革，审批时限大幅压缩。市行政服务中心投入使用，行政村综合服务大厅实现全覆盖。整合优化12345服务平台，完成网上办事大厅中山分厅镇区站建设。商事登记制度改革成效明显，新登记各类市场主体5.95万户。实施定向财力转移支付改革。国企混合所有制改革取得阶段性进展，16家市属国企开展股权招商，中山公用成功引进复星高科参股。在全省率先建立社会征信和金融服务一体化系统。农村土地承包经营权确权登记颁证试点扎实推进。基本建成农村集体资产监管交易平台，完成资产交易1770宗。教育综合改革迈出实质性步伐，9所普通高中收归市级统筹办学。中山港口岸扩大对外开放获得国务院批复，神湾港获准设立国内首个对外开放游艇码头。海峡两岸中山论坛落户中山。

　　（四）城市建设管理不断加强。翠亨新区基础设施建设加快推进，与澳门共建粤澳全面合作示范区，成为海峡两岸交流基地。深中通道上报国家发改委申请立项，我市极力争取的A3方案通过国家发改委组织的工可报告专家评审会。105国道跨线桥全线通车，广珠中线二期、纵四线等工程顺利推进，沙港公路开工建设，19条重点断头路基本打通，129条农村公路完成硬底化，危桥改造基本完成。处置闲置土地1.8万亩。“三旧”改造向纵深推进，新增实施改造土地面积2223亩。投入16亿元开展1267项秀美村庄项目建设，农村环境得到改善。完成中心城区核心区主干道雨污分流主体工程，12个镇区生活污水处理厂二期建设加快推进。三大组团垃圾综合处理基地建设和扩容项目稳步实施。建成北台、金钟山等8个森林公园、湿地公园，启动紫马岭公园、彩虹绿洲公园改造建设。淘汰“黄标车”及老旧车辆4.2万辆。空气质量优良天数创近年新高。

　　（五）城乡居民生活持续改善。十件民生实事完成。成功入选人民网“全国民生典范城市”。预计居民人均可支配收入32847元，增长8.6%。城镇新增就业5.6万人，城镇登记失业率为2.26%，农村劳动力技能培训转移就业考核排全省第二。城镇社会保险参保919.1万人次，养老待遇显著提高。全面实施大病医疗保险制度和困难居民重特大疾病医疗救助制度。社区卫生服务中心实体化建设取得阶段性成效，平价药包在基层医疗机构全面推广。低保标准提高到533元，发放低保资金4423万元，基本实现应保尽保。基本建成保障房1584套，改造250户双低家庭危房和140户优抚对象危房。4000名异地务工人员获得积分入户资格，1.2万名异地务工人员子女获得入读公办学校资格。办理老人免费乘车卡3.4万张，建成8家居家养老服务中心。成为全省第三个推进教育现代化先进市，本科录取率排全省第一。建成53个社区体育公园、40个自然村健身苑点。首条快速公交示范线投入运营，新增公共自行车6000辆。市、镇、市场三级食品检测体系加快建设，所有镇区建成食品综合快检中心。对口帮扶和扶贫工作有序开展。

　　（六）社会综合治理不断创新。积分制管理项目获全国地方政府创新奖，673名异地务工人员被聘为村（居）特别委员，40个异地商会成立“新中山人服务中心”。成功入选首批全国社会工作服务示范地区，成为国家基层综合性文化服务中心试点市。建成286个社区综合服务大厅，社区建设“2+8+N”模式入选全国创新社会治理最佳案例。创建“无三害”城市取得明显成效，禁毒工作走在全国前列，开展涉枪涉车等“六大战役”严打了犯罪，“平安校园”、“平安医院”创建达标率100%，治安视频监控系统实现村居全覆盖，刑事发案下降近两成。有效防控人感染H7N9禽流感、登革热、埃博拉出血热。形成“1+30”人才政策体系，人才集聚效应明显。所有镇区设立法律服务大厅，“一村居一法律顾问”全面铺开。信访制度改革成效明显，越级上访大幅下降。矛盾化解取得积极成效，社会大局和谐稳定。安全生产、应急管理、外事侨务、港澳事务、台务、民族宗教、统计、审计、国防建设、妇女儿童、残疾人、红十字会、气象、人防、档案、方志等各项工作取得新成绩。

　　过去一年，我们始终注重加强政府自身建设。深入开展党的群众路线教育实践活动，认真查找解决“四风”等突出问题，切实转变工作作风，努力把“为民、务实、清廉”贯彻到政府工作的方方面面。严格落实中央八项规定，大力精简会议、文件。坚持厉行节约，严格“三公”经费管理。推进依法行政，加大规范性文件清理和监管力度，完善重大行政决策合法性审查机制，在全省依法行政考评中获优秀等次。推进行政处罚自由裁量权标准化管理，行政复议委员会机制进一步完善。自觉接受人大的工作监督、政协的民主监督，共办理人大代表建议144件、市政协提案234件，满意和基本满意率达100%。深入推进政府绩效管理，加大重点政务督查力度，加强网络问政，推进政务公开，提升政府工作效能。强化监察审计，加强廉政建设。

　　过去一年，我们紧紧围绕“深化改革年”，把握改革的窗口期，坚持问题导向，全面推进经济、社会、文化、生态、民主法制等领域改革，以改革求突破，向改革要红利，在全面深化改革中推动各项工作取得新成效。我们紧紧围绕“项目落实年”，着眼于大项目、大企业、大平台、大交通，着力破解征地拆迁、国土规划、立项审批、资金紧缺等难题，在工程推进、项目落地上取得新突破。我们紧紧围绕“效能提升年”，加强制度建设，开出权力清单和责任清单，促进审批提速、行政提效、服务提质，在营商环境建设方面取得新进展。

　　各位代表，回顾过去一年，我们深深感到，在当前严峻复杂的形势下，取得这样的成绩十分不易。这是省委、省政府和市委正确领导的结果，是市人大及其常委会和市政协监督支持的结果，是全市干部群众团结奋斗的结果，是海内外各界关心帮助的结果。在此，我代表市政府，向全市广大工人、农民、知识分子、企业家、干部群众，向各民主党派、工商联、各人民团体、无党派人士和社会各界人士，向驻我市解放军和武警官兵、人民警察，向所有关心支持中山发展的老领导、老同志、港澳台同胞、海外侨胞和国内外友人表示衷心感谢！

　　在看到成绩的同时，我们也清醒地认识到，我市正处于改革攻坚和转型升级的爬坡阶段，深层次矛盾不断凸显，面临着许多困难和问题，主要是：传统的竞争优势逐渐弱化，新的经济增长点正在培育；由于多方面因素影响，经济下行压力较大，生产总值、出口等指标增速未能实现年度预期目标；经济社会发展的体制机制呈固化趋势，新旧矛盾相互交织；资源短缺与低效利用并存，制约项目落地的短板没有根本性改变；城镇化未能同工业化很好地融合互动，新型城镇化、新型专业镇建设任重道远；基础建设和城市管理滞后，交通、环境、医疗、社保、教育等民生工作，与百姓的期盼仍有差距；一些公职人员服务意识和履职能力有待提高，依法行政水平需要加强等。对此，我们将高度重视，采取有力措施认真加以解决。

　　　　　　　　　　　　　　　　　　　　　　　　　　　　　　　2015年工作安排

　　各位代表！2015年是全面深化改革的关键之年，是全面推进依法治市的开局之年，也是全面完成“十二五”规划的收官之年，做好今年工作意义重大。综观当前宏观形势，世界正在酝酿新的科技革命和产业变革，欧美发达国家提出了智能制造2.0、工业4.0概念，新的技术和商业模式的应用，必将带来世界经济的重新洗牌。中央正在实施“一带一路”战略，构建我国对外开放新格局；广东自贸区正式启动，全省新一轮改革创新大幕已经拉开；深中通道、深茂铁路、港珠澳大桥等重大战略通道进入预期，我市区位优势得以重构和凸显。尤其是习近平总书记关于我国经济发展新常态的重要论断，为我们指明了前进的方向。作为改革开放的先行地区，我市经济发展较早进入新常态，转方式调结构已进入轨道，正从高速增长转向中高速增长，从规模速度型粗放增长转向质量效益型集约增长。

　　信心比黄金更重要。我们要正确研判形势，提振信心，因势利导，把思想和行动统一到中央对经济发展新常态的研判和部署上来，认识新常态，适应新常态，引领新常态。把今年定为“狠抓落实年”，聚精会神、脚踏实地狠抓各项工作落实。着力推动经济发展提质增效升级，努力做到调速不减势、量增质更优。要把握机遇、保持定力，做到稳中求进、好中求快。更加注重实施《珠三角规划纲要》，更加注重提高发展的质量和效益，更加注重均衡发展，更加注重改善营商环境，更加注重科技创新和技术改造，更加注重生态建设和环境保护，更加注重提高人力资本素质，更加注重提升民生保障水平，促进经济平稳健康发展与社会和谐稳定。

　　2015年政府工作的总体要求是：深入贯彻党的十八大、十八届三中、四中全会和习近平总书记系列重要讲话，以及市委十三届七次全会精神，主动适应经济发展新常态，大力推进新型专业镇建设，提高经济发展的质量和效益。坚持调整结构，推动转型升级；坚持改革攻坚，释放制度红利；坚持智慧发展，建设精品城市；坚持统筹资源，优化发展空间；坚持依法行政，推进公平正义；坚持和谐善治，增进人民福祉。在新的历史起点上，以务实求进的作风狠抓落实，奋力开创“三个适宜”更加美丽中山建设新局面。

　　2015年经济社会发展的主要预期目标是：生产总值增长8.5%左右，固定资产投资增长15.5%，社会消费品零售总额增长9.5%，出口总值增长4%，一般公共预算收入增长10%以上，城乡居民人均收入增长与经济增长基本同步，居民消费价格涨幅控制在3%左右，城镇登记失业率控制在3.5%以内，人口自然增长率控制在8‰以下，减排约束性指标完成省下达年度计划。

　　为实现上述目标，必须做好以下工作。

　　一、稳增长强后劲，夯实经济发展基础

　　托住大盘稳增长，为转方式调结构创造空间和条件。统筹用好内外两个市场，坚持高水平引进来与大步子走出去相结合，坚持扩内需与稳外需相结合，发挥好消费的基础作用、投资的关键作用和出口的支撑作用，实施更加积极的财政政策，保持经济运行在合理区间。

　　大力发展实体经济。积极推进新型工业化，打响工业大会战，建设实体经济强优城市。培育发展新兴产业集群，扶持健康医药、新能源、游艇等新兴产业发展。积极参与建设珠江西岸先进装备制造产业带，引进一批高端装备项目，培育一批重点企业，推广一批产品应用示范，支持一批核心技术创新，建设一批产业发展载体，重点发展智能制造装备、海洋工程装备、光电装备等先进装备制造业。发展软件及信息服务、研发设计、检验检测认证、科技成果转化等生产性服务业。鼓励企业兼并重组，推进“个转企、小升规、规上市”。千方百计减轻企业负担，促进各类经营主体健康发展。深化市属国企混合所有制改革，支持镇区盘活镇属资产。引导现代农业示范园区建设，培育新型职业农民，探索推广“龙头企业+合作社+家庭农场”运作模式。鼓励龙头企业发展生鲜电商，重构现代农业产业生态。

　　发挥投资带动作用。推进重点项目建设，安排市重点建设项目113项，年度计划投资376.9亿元。坚持全市招商“一盘棋”，整合招商资源和政策，建立市镇两级客商服务队伍。实施靶向招商、专业招商，突出城市整体形象推介，引进一批产业链的关键环节和重点领域项目。狠抓亿元以上项目建设，推广定期会办、联合审批经验，研究实施项目投资“直接落地制”。加大基础设施投资，重点投向交通、市政、能源、水利等领域。建立投资项目事前、事中、事后管理制度，严格执行项目投资强度、节能、环保等准入标准，提高投资效益。推动政府与社会资本合作，鼓励更多社会资本参与公共领域建设。

　　积极扩大消费需求。健全职工工资正常增长机制、工资集体协商制度，提升居民消费能力。拓宽农民增收渠道，释放消费潜力。培育消费新增长点，扩大信息、养老、健康、文化体育等新型消费业态，适度开发高端消费。推进省级旅游综合改革示范市建设，加强旅游规划统筹，整合旅游资源，培育旅游新业态和新产品，扩大旅游综合消费，拓展国内外旅游合作领域。加强商业网点规划，鼓励连锁企业进社区，统筹城市综合体、大型商业广场和镇区商贸中心建设。规范市场秩序，优化消费环境。要立足当前和考虑长远，保持房地产政策相对稳定，进一步清理和规范相关收费，积极消化存量，科学控制增量，引导房地产业健康有序发展。

　　提升对外开放水平。争取中山港、神湾港口岸扩大对外开放年内通过验收，开通黄圃港口岸。加快发展海洋经济。加强与港澳合作，实现中山-澳门游艇自由行。深化口岸通关改革，推进电子口岸系统建设与省内区域通关一体化。积极申报跨境电子商务试点城市，推进市级跨境贸易电子商务园建设。扶持发展外贸综合服务企业，发展旅游购物、市场采购等外贸新模式，设立市场采购出口商品监管区。发展服务贸易。培育外贸品牌和境外营销网络，支持中山产品进入国际组织采购名录。推进保税物流中心、华南进口乳品交易中心建设，打造红木、大宗消费品等商品进口新平台。促进加工贸易转型升级，帮助企业拓展内销市场。加强与省自贸区对接与合作，研究创建综合保税港区。积极参与“一带一路”建设，大力拓展东盟、中东、拉美等新兴市场。鼓励企业“走出去”。

　　支持欠发达镇区提速发展。推动区域均衡发展，加大对欠发达镇区的扶持力度。新增4.9亿元用于镇区定向财力转移支付，安排4.2亿元用于欠发达镇区均衡性转移支付。统筹安排1亿元用于镇区生态补偿横向转移支付。安排4.5亿元支持欠发达镇区园区基础设施建设。

　　二、加快转型升级，激发经济内生动力

　　发挥市场在资源配置中的决定性作用，通过更大地释放改革的活力，更多地依靠创新的力量，来激发内生动力，更加突出经济发展的科技含量和生态质量，让发展的质量更高、效益更好、后劲更足。

　　大力推进战略平台建设。推进新型专业镇发展，实施八大创新工程，打造“两区四带多集群”产业格局。积极争取和用好省的政策支持，加快翠亨新区建设。推进中山港东移、翠亨新区路网桥梁等重大基础设施建设，翠亨快线年内通车。启动翠亨新区科技金融新城建设。高水平建设粤澳全面合作示范区和海峡两岸交流基地，推进一批优质项目落地。推进四大市级产业平台建设，鼓励各镇区向平台输送项目。

　　加强科技创新和技术改造。以创新驱动发展为抓手，构筑城市创新空间集聚集约，技术与产业创新协同协作的创新生态。落实加速折旧等措施，支持企业升级设备，推动“机器换人”和“智能化车间”建设。优化财政科技资金的投入方式，探索发行科技创新券。设立市重大科技专项投资基金。深化产学研合作，推进工业发展研究院建设，提升市生产力促进中心联盟服务功能。建设国家重点实验室等高层次创新平台，培育大中型企业研发中心和科技企业孵化器，鼓励发展各类新型研发机构。加快组建“大仪网”，实现科技服务“云”共享。加强知识产权保护和利用。

　　增强金融的供血能力。做好城乡金融服务一体化综合改革试点。完善中小微企业金融服务体系，促进融资性担保行业发展。推广建设专业镇金融服务中心，完善专业镇金融超市与服务平台。发挥好100亿元市建设发展股权基金作用，探索设立新兴产业创投引导基金，鼓励企业境内外上市和新三板挂牌。发展社区金融、互联网金融、消费金融等新型金融模式。完善地方金融生态环境，防范和化解金融风险。

　　培育发展新型商业模式。新型商业模式是一片沃土，政府要培土施肥、精耕细作。加大力度扶持电子商务发展，实施电子商务发展规划。加快发展电子商务新兴业态，支持电子商务园区、专业市场电子商务平台、社区电子商务平台建设。大力培育和引进一批龙头电商企业和行业电商平台，鼓励发展线上线下结合、移动互联等业务，加强与国内外大型电商平台合作。支持开展基于大数据的开发研究和整合运用，加快完善大数据生态产业链。创建“中山美居”网络品牌，建设“中山美居”体验平台。整合装饰行业资源，打造泛装饰交易平台。

　　集聚服务创新创业人才。建设国家级留学人员创业园，引进高层次人才入园创新创业。扶持创新科研团队，落实高层次人才入户、子女入学等政策。实施新型企业家培养工程，选拔一批优质企业的经理人进行培训。创新高层次人才评定，培养企业“双高”人才。加大实用型人才的引进力度，推进专业技术人员职称制度改革。加强劳动者培训，实施劳动力技能晋升培训1.5万人，提高劳动者技能素质。办好“中山人才节”和高层次人才联谊会，营造良好的选人用人环境。

　　三、优化发展空间，推动城市扩容提质

　　把扩容提质作为城市升级的战略方向，放大格局谋划城市发展，按照产城融合、智慧人本的理念，提升城市承载力，按照精心规划、精品建设、精细管理的要求，打造经济发达、功能完善、环境优美、特色突出、文化浓郁的现代精品城市。

　　加快推进“三规合一”。突出规划引领作用，提高政府对空间的管控能力。年内完成“三规合一”规划编制，形成一本规划一张蓝图。严格划定生态控制线，科学界定城市开发边界。统筹土地管理，开展农村地籍调查工作。规范地下空间利用。用实事求是、勇于担当的精神解决历史遗留问题。全面科学谋划和编制好“十三五”规划。

　　提高土地利用综合效益。开展建设用地节约集约评价工作，推动工业用地竞争供给，建立低效利用土地退出机制。盘活存量土地，加大存量建设用地收储力度，完成存量建设用地清查工作。加快“三旧”改造，推进闲置用地处置，铁腕整治“两违”问题。有序发展商品厂房，推动低开发强度厂房改造，鼓励“企业上楼”。通过扩、消、并、转，整合和提升工业园区，促进园区二次开发。

　　加快新型城镇化建设。实施“强心优副”战略，提高中心城区集聚能力，强化小榄、三乡副中心公共设施建设。围绕综合交通枢纽站场，推行公交导向型开发模式。建设智慧城市，加快电子政务、北斗等云平台建设。推进社区“数字化”建设，建立社区管理信息化平台和基础数据库。打造人文中山，推进岭南文化特色街区复兴，加强对古街区的保护和利用。

　　大力推进交通路网建设。坚持交通先行，积极配合做好深中通道前期工作，推动深中通道按照A3方案年内动工建设，加快推进配套路网对接工程建设。争取广中珠澳城际纳入珠三角城际轨道网规划，推动深茂铁路深圳至江门段尽早建设。推进城市轨道交通前期工作，构建中山捷运系统。加快中开高速及小榄支线、广中江高速建设。完善骨干交通网络，建成阳光大道接福源路、十水线小榄水道特大桥，开工建设岭栏路、二环快速路港口至横栏段、番中公路改建工程，续建纵四线、大南路、古神公路二期、广珠中线二期，改造岐江公路沙溪段、长江路、省道S268线古镇至横栏段。加大中心城区道路网络改造建设力度。增建公交站场，完善自行车、行人慢行交通系统。启动农村公路建设计划，推进主干农路硬底化二期工程。

　　增强城市保障能力。加强能源供应的调度和储备，加快热电联产、天然气管网等项目建设。完善通信和供电网络，开展“宽带中国”试点城市建设。完善防洪排涝体系。推进抗咸工程建设。推进水利现代化试点工作，加快高标准基本农田建设。推进气象现代化建设，提升防灾减灾能力。

　　四、加强生态建设，打造更加宜居美丽中山

　　坚持绿色低碳的发展理念，守住生态底线，加强污染治理、环境整治和生态修复，创造更多绿色财富，守护天更蓝、地更绿、水更清的美好家园。

　　扩大绿色空间。落实生态文明建设规划，加快全国生态文明示范区建设。推行低冲击开发模式，加强对城市绿地、山体、河流、岸线的开发管理。实施生态修复工程，推进森林进城、森林围城，建设城镇景观林带和环城防护林，建设一批公园。支持岐江河沿线镇区打造滨水走廊。推进秀美村庄建设，大力整治农村人居环境。

　　加强环境保护和治理。加大内河涌整治力度，逐条消除中心城区内河涌劣五类水体。继续推进城乡雨污分流，加强岐江河水环境综合治理。加快整治西江中山河段、小榄水道、鸡鸦水道等饮用水源二级保护区。推进农产品产地土壤重金属污染修复。开展大气污染防治，实施重点企业在线监控，加强燃煤扬尘、尾气废气和挥发性有机物治理，加快淘汰锅炉和“黄标车”。建立最严格的用水用地管理制度，加强资源能源利用效率评价。

　　大力推进节能减排。抓好重点领域、重点行业节能减排，推进工业、建筑、交通、公共机构和居民生活节能。推广LED绿色照明和新能源汽车，培育发展节能环保产业。加强重点用能企业监管，推进集中分布式能源项目建设。鼓励企业清洁生产。统筹环境容量指标管理，加快建立节能量、排污权和水权交易制度。严把环保准入关口，杜绝重污染项目上马。继续实施差别水电气价格，加快淘汰落后产能。

　　五、改善保障民生，提升人民群众幸福感

　　坚持把人民群众放到心中最高位置，牢牢树立发展依靠人民、发展为了人民的理念，更加注重满足人民的需要，更加注重保障基本民生，努力让百姓生活更加体面和幸福。

　　构建普惠托底的社会保障。市财政统筹安排9.4亿元，用于生活、住房、医疗等社会保障。健全养老、生育、工伤保险制度，扩大特定病种医保支付范围。实施创业带动就业工程，帮扶高校毕业生等重点群体就业创业。完善社会救助体系，提高最低生活保障标准，推进低保、低收入家庭危房改造。实施困难居民重特大疾病医疗救助。建立社会力量参与防灾减灾机制。加快保障性住房建设，新建公租房1557套，基本建成1800套。推进慈善、残疾人、红十字会等事业改革发展。

　　办人民满意的教育。制定实施第二期学前教育三年行动计划，新增规范化幼儿园学位2000个。推进义务教育学校标准化建设，促进义务教育均衡发展。推动普通高中集约化发展，提高优质普通高中指标生比例。探索家庭教育新形式，促进孩子健康成长。创新公办职业学校办学模式，深入推进职业教育产教融合、校企合作，探索社会资本办学与职教联盟新机制。改造提升特殊教育学校。促进民办教育规范特色发展。推进省市共建研究生联合培养基地建设。

　　提供更优质的医疗卫生服务。完善社区卫生服务中心功能和运行机制，扩大家庭医生式服务和“医联体”试点范围。健全基层首诊、双向转诊制度。鼓励医生多点执业。完善中医预防保健网络，推动中医药进社区。鼓励社会办医，构建多元办医格局。深化平安医院建设。做好疾病防控工作，及时有效应对各类突发公共卫生事件。加强职业危害管理，保障劳动者健康。

　　加强和创新社会治理。完善“全民参与”社会治理机制，探索社会治理中山模式。启动综合网格化管理改革试点，探索建立“一法五全”的网格化治理体系。加强社区综合服务大厅规范化建设，实现社区建设“2+8+N”模式高标准广覆盖。加大政府购买社会服务力度，强化服务质量监管。建立社会组织综合监管体制，提高社会组织承接外包服务能力。扩宽异地务工人员参与社会治理的渠道。推进不动产统一登记。健全“大调解”工作格局，完善调解、仲裁、诉讼等多元化纠纷解决机制，推进网上信访工作。加强劳资关系源头治理。健全公共法律服务体系，提升社会治理法治化水平。

　　维护和保障社会安全。深化平安中山建设，大力开展全民治安，提升“平安细胞”创建的广度和深度。巩固提升医院“无医闹”、场所“无三害”、社区“无毒害”、城市“无三非”工作，创新立体化社会治安防控体系。加大力度推进“平安出租屋”建设，推动刑事犯罪案件明显下降。开展社区戒毒康复，推进社区矫正和安置帮教工作。加大食品药品安全监管力度，构建社会共治格局，创建无假药城市。推进家禽集中屠宰、冷链配送、生鲜上市。推进动物无害化处理厂建设，加强重大动植物疫病防控。加强安全生产，强化应急管理。做好公共安全工作。

　　全面发展各项社会事业。持续开展全民修身行动。推进中山纪念图书馆、139文化街区等重大文化基础设施建设。推进国家基层综合性文化服务中心试点工作。完善公共文化服务体系，鼓励更多社会资本参与文化建设。推进传统媒体与新媒体融合发展。办好孙中山文化节，筹备孙中山诞辰150周年纪念活动。擦亮“中山杯”华人华侨文学奖等文化品牌。加强文化遗产的传承保护。支持发展体育产业，广泛开展全民健身活动。创建全国“双拥”模范城。加强国防建设，做好人口计生、外事侨务、港澳事务、台务、民族宗教、气象、统计、妇女儿童、人防、地震、档案、方志等工作。做好对内对外扶贫工作。

　　六、扎实办好实事，切实增进民生福祉

　　坚持包容性发展，发挥社会政策托底作用，实施“市民点菜、政府埋单”，全力办好一批民生实事，努力把民生实事办到百姓的身边，办到百姓的心坎上。

　　经过广泛征集民意，今年继续集中力量办好十件民生实事。一是群众出行“最后一公里”畅通工程。完成新建、改建村级公路项目830个，里程约324.9公里，解决167个行政村群众出行难问题。二是放心食品工程。加强对食品种养、流通、消费环节的快检快筛，全市快检农产品、水产品、餐饮食品50万份。完善镇区水产品快速检测点24个。建成动物卫生远程视频，对全市畜禽定点屠宰企业和批发市场进行实时监控。建成市总控中心1个、镇分控中心24个、重点企业监控点203个。实现200亩以上蔬菜基地产品安全检测全覆盖，新增放心菜生产基地1000亩。三是放心饮水工程。对城区3万户用水户自来水管网进行改造，将易锈蚀的灰口铸铁管和镀锌钢管更换为耐腐蚀的聚乙烯管等管材，增加管网末梢定期排水，管网水质综合合格率达99%以上。四是便民就医工程。在市区域医疗卫生信息平台和实名制基础上，实现市人民医院、市中医院、市博爱医院、小榄人民医院等三甲医院检验检查结果和诊疗信息互联互通，实现三甲医院微信、网站、电话等多渠道预约，方便群众就医。五是城市“绿肺”工程。紫马岭公园增加夜间开放时段，田心、金钟山、北台等3个森林公园对外开放，丫髻山、铁炉山、银坑等3个森林公园及树木园三期完成规划设计并启动建设。建成西区彩虹绿洲公园、港口湿地公园，推进翠亨新区国家湿地公园和五马峰公园建设。绿化美化村庄40个，森林景观改造2500亩。六是内河清流和城区治涝工程。在完成中心城区核心区主干道雨污分流主体工程基础上，开展首批内河涌整治，年内治理主城区发疯涌、白石涌、西河涌、崩山涌、羊角涌5条内河涌和镇区茅湾涌等48条内河涌约300公里，年底前基本消除这些河涌劣五类水体、“黑臭”现象和垃圾漂浮问题。此外，对旧有排水系统启动升级改造工程，对兴中道、华柏路、博爱七路、悦盈新城、起湾道（起湾道与中山四路交叉口）及兴华大街（兴华大街与建设街交叉口）等6个内涝易发点进行排水改造。编制完成《中山市城市排水（雨水）防涝综合规划》。白石涌排涝泵站投入使用，建成发疯涌排涝泵站。七是创业带动就业工程。新增创业园区（孵化基地）6个，组建50人创业导师团指导创业，扶持500名有创业愿望人员入园孵化，1.5万人接受创业教育。新增市场主体8000家，城镇新增就业5万人。八是居家养老服务中心全覆盖工程。居家养老服务中心覆盖全市所有镇区，为老年人提供日间照料、文体娱乐、康复保健、辅导培训、助餐等居家养老服务。九是全民健身工程。新建全民健身驿站60个，形成城乡居民15分钟步行健身圈。100所公办学校体育场馆向公众开放。每个镇区举办12场次群众性体育赛事活动。十是出租屋平安工程。落实出租屋备案登记，实行分层分类服务管理。实施出租屋网格化管理，推行网格内“联合巡查、综合执法”。推进出租屋信息化管理，在全市出租屋推广应用门禁视频系统，年内覆盖50%规模以上出租屋。

　　各位代表！做好2015年各项工作，必须不断加强政府自身建设，以更加优良的作风、更加勤勉的工作、更加高效的服务，建立权责统一、务实高效的依法行政体制，建设法治政府和服务型政府。

　　一是坚持依法行政。全面落实市镇两级政府法律顾问制度，提高干部运用法治思维和法治方式履行职能、推动改革的能力。严格执行重大行政决策程序管理制度，落实重大行政决策风险评估、合法性审查等程序规定。建立规范性文件正面清单管理制度。完善市镇权责清单，建立审批服务事项目录管理系统。深化行政处罚自由裁量权标准化建设，严格行政执法人员持证上岗管理，推广镇区综合执法模式。扩大政务公开领域和范围。完善行政复议机制，妥善化解行政争议。

　　二是坚持奋发有为。以昂扬向上的精神状态狠抓落实。树立正确的激励导向，为想干事、能干事、干成事的人提供舞台、创造条件，为创新和探索留出足够的空间。加大培训力度，完善领导干部知识结构，推动知识能力转型升级，提升公职人员适应新常态、把握新形势、驾驭新局面的能力，打造一支高素质干部队伍。

　　三是坚持优化服务。巩固党的群众路线教育实践活动成果，坚持“开门抓作风”，建立密切联系群众的常态化工作机制。深化行政审批体制改革，落实“三集中一分开”。完善市行政服务中心功能，提升网上办事大厅业务办结率。规范建设项目中介组织管理，建设网上“中介超市”。探索推行“三证合一”改革，推进工商登记全程电子化。清理涉企行政事业性收费，减轻企业负担。增强政府网站服务功能，探索政务服务微平台，健全线上线下政务服务体系。加强督办，深化督考合一模式，实现绩效管理镇区全覆盖。加强行政效能监督，自觉接受人大、政协、社会各界的监督，主动听取各民主党派、工商联、人民团体和各界人士的意见建议。

　　四是坚持廉洁自律。坚持把权力关进制度的笼子，落实“一岗双责”，探索建立重大工程项目廉情预警系统。完善内部审计“年审制”，突出对改革措施、财政资金、政府重点投资项目、领导干部经济责任、国有资产、国有资源等方面的重点审计，加强对农村集体资产和村级财务专项审计的监督。逐步推进镇区集中支付改革。严格落实八项规定和廉政建设各项规定，严格执行公务接待、因公出国（境）、公车使用等规定，严格控制“三公”经费、楼堂馆所建设和人员编制增长。

　　各位代表！新的一年，适应新常态，要有新气象。人民的利益，是我们一切工作的出发点和落脚点；人民对美好生活的追求，是我们的奋斗目标；人民的嘱咐重托，是我们的神圣使命！让我们在中共中山市委的领导下，励精图治，奋发有为，带着“精品城市、和美中山”的美好追求，拼搏，进取，再创业；革新，突围，再崛起！
